# ASPREE SUB-STUDIES

**DOI:** 10.1093/geroni/igz038.2361

**Published:** 2019-11-08

**Authors:** Robyn L Woods, John J McNeil, Anne Murray

**Affiliations:** 1 Monash University, Melbourne, Victoria, Australia; 2 Berman Center for Outcomes and Clinical Research, Minneapolis, Minnesota, United States

## Abstract

To maximize opportunities provided by a large-scale clinical trial, we established sub-studies during the ASPREE trial to investigate specific areas of interest to the health of older persons. A biobank now stores multiple aliquots of blood, urine or saliva collected from >15,000 healthy ASPREE participants across the US and Australia, most at baseline (pre-randomization) and/or after 3 years post- randomization. Other sub-studies included ALSOP (ASPREE Longitudinal Study of Older Persons; sets of questionnaires administered every 2 years focused on medical or social issues) and neuroimaging projects with brain MRI and retinal vascular imaging to investigate anatomical changes linked with cognitive or cerebrovascular outcomes. Further sub-studies addressed the impact of aspirin on cerebral microhemorrhages, age-related macular degeneration, age-related hearing loss, severe sepsis, and falls and fractures. Biomarker analyses underway include plasma androgens in older women and DNA sequencing of the cohort to investigate contributions of genomics to aging health and disease.

